# Impact of nurse-led supportive care intensity on quality of life and symptom burden in patients undergoing palliative chemotherapy: A prospective cohort study

**DOI:** 10.1097/MD.0000000000049780

**Published:** 2026-07-24

**Authors:** Bao-Mei Wang, Wen-Ling Wang, Yong-Jian Zhang, Meng Wu, Hai-Xia Wei, Shu-Yan Zhang, Meng Li, Bo-Bo Yan, Wen Zhang

**Affiliations:** a Affiliated Hospital of Hebei University of Engineering, Handan, Hebei Province, China.

**Keywords:** advanced cancer, coping, nurse-led care, palliative oncology, quality of life, self-management, supportive care intensity, survival, symptom burden

## Abstract

Nurse-led supportive care is integral to palliative oncology, yet the intensity of nursing contact is rarely quantified, and its clinical relevance remains incompletely defined. We prospectively examined the association between supportive care intensity (SCI) and patient-reported outcomes and survival in patients receiving palliative chemotherapy. This prospective observational cohort study enrolled adults with advanced solid tumors undergoing palliative chemotherapy. All participants received routine physician-led oncology care and standard chemotherapy nursing, while additional supportive services were delivered according to usual clinical practice and were not protocolized as part of the study exposure. Patients were followed for 24 weeks for patient-reported outcomes, with survival follow-up until data lock. SCI was quantified based on the frequency and depth of nurse-delivered supportive care encounters, encompassing symptom assessment, patient education, coping support, self-management coaching, caregiver engagement, and remote follow-up. Outcomes included quality of life (European Organization for Research and Treatment of Cancer QLQ-C30), symptom burden, coping (Brief COPE), self-management capability, existential well-being, and overall survival. Linear mixed-effects models and Cox proportional hazards models were applied with adjustment for key clinical and demographic covariates. A total of 180 patients were included. Baseline demographic, clinical, and patient-reported characteristics were generally similar across SCI quartiles, although modest imbalance was observed in selected clinical variables, particularly Eastern Cooperative Oncology Group performance status. Higher SCI was associated with significantly greater improvements in global QOL over 24 weeks (*P* < .001), showing a graded association across SCI levels. Improvements were most pronounced in physical and role functioning, while fatigue, appetite loss, and constipation showed the largest symptom reductions. Higher SCI was associated with a greater likelihood of achieving a clinically meaningful ≥10-point improvement in global QOL (adjusted odds ration [OR] 1.46, 95% confidence interval [CI] 1.12–1.90), lower Edmonton Symptom Assessment System scores (β − 2.21, *P* < .001), more adaptive coping strategies, higher self-management capability (adjusted OR 1.82, *P* < .001), and better existential well-being. In exploratory survival analyses, higher SCI was associated with lower adjusted mortality risk (adjusted hazard ration [HR] 0.68, 95% CI 0.48–0.96), with a possible contact-frequency pattern observed for ≥8 nurse-led supportive care encounters (adjusted HR 0.61, *P* = .018). Within routine oncology care, greater intensity of nurse-led supportive care was associated with multidimensional improvements in QOL, symptom burden, coping, and self-management, and showed a favorable exploratory association with lower adjusted mortality risk.

## 1. Introduction

Advanced cancer imposes a substantial and multidimensional burden on patients.^[1,2]^ Physically, individuals frequently experience clusters of distressing symptoms, including pain, fatigue, nausea, and dyspnea, which often coexist and worsen over the disease trajectory.^[3,4]^ Psychological distress, including anxiety, depression, and fear, is common and further complicates adaptation to illness.^[2,5]^ Social functioning declines as patients lose work capacity and face growing family responsibilities, while existential concerns related to loss of control and fear of death frequently emerge.^[4,6,7]^

Conventional anticancer treatments cannot fully address these needs. Tumor-directed therapies often overlook the patient’s overall experience, and limited clinical resources can lead to inadequate symptom assessment and management.^[8,9]^ In many settings, comprehensive supportive care systems remain underdeveloped.^[10]^ Consequently, patients face persistent unmet needs, including insufficient symptom control, limited communication and information support, inadequate coping skills, and a reduced sense of control over quality of life (QOL) and prognosis.^[11,12]^

International guidelines strongly support early integration of comprehensive supportive or palliative care (end-of-life palliative/palliative supportive care).^[13,14]^ Early palliative care has been shown to enhance QOL, reduce symptom burden, improve psychological well-being, and promote more adaptive coping.^[15–17]^ In certain cancer types – most notably advanced lung cancer – it has even demonstrated survival advantages.^[13]^ Nurse-delivered interventions are central to this model. Nurses are the professionals who maintain the closest and most continuous contact with patients, playing key roles in symptom monitoring, patient education, and psychosocial support.^[15,16]^ Increasing evidence indicates that nurse-led supportive care approaches are both feasible and effective.^[18,19]^

Despite strong evidence for early palliative and supportive care, key gaps persist. The intensity of nurse-led supportive care has rarely been quantified, as prior studies typically compare intervention versus no intervention and overlook variation in the frequency and depth of nurse–patient contact.^[20–22]^ Consequently, how supportive care intensity influences QOL, symptom burden, or potential dose–response effects remains unclear, and evidence for survival impact is minimal.^[23–25]^ These gaps underscore the need for prospective real-world studies that systematically evaluate supportive care intensity and its outcomes.

Conceptually, baseline patient need may influence both the likelihood of receiving higher-intensity nurse-led supportive care and subsequent outcomes, whereas supportive care intensity may be associated with outcomes through intermediate changes in symptom burden, coping, self-management capability (SMC), and existential well-being. This prespecified framework informed the study design and analytic approach.

From a methodological perspective, this study is not intended as a measurement development or comparative effectiveness trial. Instead, supportive care intensity (SCI) is treated as an exposure proxy reflecting real-world variation in nurse-led supportive care processes within routine oncology practice, with potential relevance for quality assessment and future implementation efforts rather than as a prespecified intervention target.

Therefore, this study aims to measure supportive care intensity and assess its associations with QOL, symptom burden, coping, self-management, and overall survival in patients receiving palliative chemotherapy. Clarifying these relationships will help define the independent contribution of nursing intensity and strengthen the evidence base for nurse-led supportive care.

## 2. Methods

### 2.1. Study design

This study employed a prospective observational cohort design. A total of 180 patients were included. Patients were consecutively recruited from the Department of Oncology at Hebei Engineering University Affiliated Hospital between January 2021 and December 2023. The data-lock date for all analyses was January 31, 2024. The sample included all eligible patients who met the inclusion criteria and provided written informed consent.

Each participant was followed for 24 weeks to assess patient-reported outcomes, with survival follow-up continuing until the final data-lock date. Loss to follow-up differed by outcome. For survival ascertainment, 18 patients (10.0%) were lost to follow-up and were censored at the date of last confirmed contact. Attrition for longitudinal patient-reported outcome assessments reached approximately 20% over the 24-week follow-up period, primarily due to disease progression, clinical deterioration, or voluntary withdrawal.

Expected survival of at least 3 months was assessed at enrollment by the treating oncologist based on routine clinical judgment, incorporating disease stage, planned chemotherapy regimen, performance status, and prior treatment response. Written informed consent was obtained from every participant before data collection. The study protocol was reviewed and approved by the hospital’s Institutional Ethics Committee and was conducted in accordance with the Declaration of Helsinki.

### 2.2. Inclusion and exclusion criteria

Participants were eligible for inclusion if they met the following criteria: age ≥18 years; histologically or radiologically confirmed advanced solid tumor; currently receiving palliative chemotherapy; Eastern Cooperative Oncology Group (ECOG) performance status of 0 to 2; an estimated life expectancy of at least 3 months; ability to complete patient-reported outcome questionnaires, including the European Organization for Research and Treatment of Cancer (EORTC) QLQ-C30, Edmonton Symptom Assessment System (ESAS), Brief COPE, and SMC scales; and provision of written informed consent.

Participants were excluded if they met any of the following criteria: First,significant cognitive impairment or inability to communicate effectively, preventing completion of assessments. Second, concurrent participation in any other interventional clinical trial. Third, severe psychiatric disorders (e.g., uncontrolled depression and psychosis) that could interfere with study participation. Fourth, incomplete clinical data or inability to complete follow-up assessments.

### 2.3. Nurse-led supportive care model

#### 2.3.1. Construction of the SCI index

SCI was defined as a multi-component measure capturing the frequency and depth of nurse-led supportive care delivered during routine palliative chemotherapy. The index encompassed several core dimensions of nursing practice: symptom assessment performed at least once per treatment cycle; evidence-based symptom education; coping-enhancement counseling, typically provided through 3 or more structured sessions when indicated; self-management coaching targeting daily symptom control and treatment adherence; caregiver involvement or care coordination to support home-based management; telephone or remote follow-up contacts; and the total number of nursing encounters.

Each completed nursing activity was assigned 1 point, with additional points awarded based on the frequency of encounters. The cumulative score generated a continuous SCI measure ranging from 2 to 13, reflecting increasing levels of supportive care engagement.

#### 2.3.2. SCI classification

For primary analyses, SCI was categorized into quartiles (*Q*1–*Q*4), representing increasing levels of supportive care intensity from lowest to highest. A binary classification was further applied to compare high-intensity care (*Q*4) with low-to-moderate intensity care (*Q*1–*Q*3).

#### 2.3.3. Sensitivity and dose–response analyses

To explore potential dose–response patterns, a threshold analysis was performed using ≥8 nursing encounters as an indicator of high-intensity care, compared with <8 encounters. This classification was used in sensitivity analyses to assess the robustness of associations between SCI and clinical outcomes.

#### 2.3.4. Operationalization and standardization of SCI components

Supportive care activities contributing to the SCI index were prespecified prior to study initiation and operationalized using standardized definitions. These activities included symptom assessment, patient education, coping and psychological support, self-management coaching, caregiver engagement, and remote follow-up. All participating nurses received structured training on SCI documentation, including orientation to activity definitions, scoring rules, and examples of eligible encounters. Nurse–patient supportive care encounters were documented prospectively using a standardized template that captured encounter modality (in-person or remote), duration, and content domains addressed during each contact. SCI scores were derived directly from these prospectively recorded data fields rather than from retrospective chart abstraction. In addition, documentation completeness and internal consistency were routinely reviewed by the study team/senior nursing staff as part of data quality control. Although formal inter-rater reliability coefficients were not prospectively estimated, these procedures were implemented to enhance the reproducibility of SCI ascertainment in routine practice.

### 2.4. Outcomes

#### 2.4.1. Primary outcome

The primary outcome was health-related QOL, measured using the EORTC QLQ-C30 questionnaire. Assessments were conducted at baseline, 12, 18, and 24 weeks. The primary indicator was the Global Health Status/QOL scale, supplemented by functional and symptom domains. A clinically meaningful improvement was defined as an increase of ≥10 points from baseline.

#### 2.4.2. Symptom burden

Symptom burden was evaluated using the ESAS, including both the total score and individual symptom items such as pain, fatigue, nausea, and sleep disturbance. High symptom burden was defined as an ESAS total score ≥30.

### 2.5. Coping ability

Coping strategies were assessed with the Brief COPE scale, covering adaptive domains such as active coping, emotional regulation, acceptance, and positive reframing.

#### 2.5.1. Self-management capability (SMC)

Self-management capability was measured across 3 domains – core strategies, preparatory strategies, and implementation strategies. High SMC was defined as a score at or above the 75th percentile.

#### 2.5.2. Existential well-being

Existential well-being was assessed using the existential subscale of the Functional Assessment of Chronic Illness Therapy-Spiritual Well-Being instrument. Scores were calculated according to the standard scoring manual, with higher scores indicating better existential well-being.

#### 2.5.3. Overall survival

Overall survival was defined as the time from study enrollment to death from any cause. Survival status was determined through medical record review and telephone follow-up. Patients lost to follow-up were censored at the date of their last confirmed contact.

### 2.6. Data collection

#### 2.6.1. Baseline assessment (*T*0)

Baseline data were collected at enrollment and included demographic characteristics (age, sex, marital status, educational level, and monthly income) and clinical information such as cancer type, disease stage, metastatic sites, ECOG performance status, and comorbidities. Patient-reported outcomes were obtained using the EORTC QLQ-C30, ESAS, Brief COPE, SMC scale, and edmonton balance (EB) measures. Caregiver involvement at baseline was also recorded.

#### 2.6.2. Follow-up assessments (*T*1–*T*3)

Follow-up patient-reported outcome assessments were conducted at 12 (*T*1), 18 (*T*2), and 24 weeks (*T*3). Overall survival was monitored continuously until the final data-lock date through electronic medical records and telephone confirmation. Participants who became unreachable were censored at their last verified contact.

#### 2.6.3. Documentation of nursing encounters

Nursing encounters were prospectively recorded by the clinical nursing team. Documentation included the frequency, content, and duration of each supportive-care contact, as well as the mode of delivery (in-person or remote). These data were used to construct the SCI index.

### 2.7. Statistical analysis

Descriptive statistics were used to summarize baseline demographic, clinical, and patient-reported characteristics. Continuous variables were reported as mean ± standard deviation or median with interquartile range (IQR), whereas categorical variables were presented as counts and percentages. Group differences across quartiles of supportive care intensity (SCI; *Q*1–*Q*4) were assessed using ANOVA or the Kruskal–Wallis test for continuous variables and the χ^2^ test for categorical variables. Standardized differences were additionally reported to evaluate baseline comparability across SCI quartiles. The distribution of SCI scores was summarized using means, standard deviations, medians, and IQRs. Frequencies of individual nursing activities contributing to the SCI index were also described.

A logistic regression model was used to identify baseline factors associated with receiving high-intensity supportive care, defined as *Q*4 versus *Q*1 to *Q*3. Covariates included age, sex, cancer type, ECOG performance status, baseline QOL, baseline ESAS score, and caregiver involvement. Results were reported as odds ratios (ORs) and adjusted ORs with 95% confidence intervals (CIs).

Longitudinal changes in QOL were analyzed using linear mixed-effects models (LMMs). Fixed effects included time, SCI, and the SCI × time interaction, while individual participants were modeled with random intercepts. Models were adjusted for age, sex, cancer type, ECOG performance status, baseline global QOL score, and baseline ESAS score. Analyses examined global QOL, functional domains, and symptom domains, with predicted trajectories plotted graphically. Logistic regression was additionally used to evaluate clinically meaningful QOL improvement, defined as a ≥10-point increase from baseline, with results reported as adjusted ORs and 95% CIs.

Total and individual ESAS symptoms were analyzed using LMMs. Models were adjusted for age, sex, cancer type, ECOG performance status, baseline ESAS total score, and baseline global QOL score. Predictors of high symptom burden, defined as ESAS ≥ 30 at follow-up, were examined using logistic regression. Changes in coping strategies assessed by the Brief COPE scale were analyzed using LMMs focusing on adaptive coping domains. Each SMC subdomain, including core, preparatory, and implementation strategies, was modeled using LMMs. Logistic regression was used to identify predictors of high SMC, defined as a score at or above the 75th percentile at follow-up. Changes in existential well-being were examined using LMMs or generalized estimating equations, depending on distributional assumptions.

For longitudinal analyses, LMMs included participant-specific random intercepts and an unstructured covariance matrix to account for within-subject correlation across follow-up time points. Models were estimated using all available observations under the missing-at-random assumption. No imputation was applied for longitudinal outcomes within the mixed-effects framework.

SCI was treated as a cumulative exposure summarizing nurse-led supportive care received during follow-up rather than as a time-updated variable. Accordingly, time-varying confounding due to escalation of supportive care in response to worsening symptoms was not explicitly modeled. Longitudinal associations should therefore be interpreted as average associations over follow-up rather than time-specific causal effects.

Overall survival was analyzed using Kaplan–Meier curves comparing high-intensity versus low-to-moderate SCI groups, with differences tested using the log-rank test. Multivariable Cox proportional hazards models were used to estimate adjusted hazard ratios (HRs) for the association between SCI and all-cause mortality, controlling for age, sex, cancer type, ECOG performance status, baseline global QOL score, and baseline ESAS total score. The proportional hazards assumption was assessed using Schoenfeld residuals. Sensitivity analyses examined a contact-frequency threshold using ≥8 versus <8 nurse-led supportive care encounters, with additional Kaplan–Meier and Cox models performed accordingly.

Exploratory pathway-related analyses examined whether higher SCI was associated with early changes in global QOL and SMC and whether these early changes were associated with subsequent mortality risk. These analyses were descriptive and were not based on a prespecified causal mediation framework, formal structural equation modeling, or assumptions required for estimating indirect effects.

Early death was defined a priori as death occurring within 30 days of study enrollment. Sensitivity analyses excluding patients who experienced early death were performed to evaluate robustness. Missing covariate data were handled using multiple imputation by chained equations. The imputation model included age, sex, ECOG performance status, cancer type, baseline global QOL, baseline symptom burden, SCI, and survival status. A total of 20 imputed datasets were generated, and parameter estimates were pooled using Rubin rules. Convergence was assessed through visual inspection of trace plots and comparison of variable distributions across imputations.

Additional sensitivity analyses included complete-case analyses, multiple-imputation analyses, exclusion of early deaths, reanalysis using binary SCI classifications, and prespecified subgroup analyses stratified by age, sex, cancer type, and ECOG performance status.

## 3. Results

### 3.1. Baseline characteristics

A total of 180 patients were included in the analysis and categorized into quartiles according to nurse-led supportive care intensity, with 45 patients in each quartile. Table 1 summarizes baseline sociodemographic, clinical, and patient-reported characteristics across SCI quartiles. Overall, baseline characteristics were generally similar across groups, although modest imbalance was observed in selected variables.

**Table 1:** Baseline sociodemographic, clinical, and patient-reported characteristics by quartiles of nurse-led supportive care intensity (SCI).

Variable	*Q*1 (n = 45)	*Q*2 (n = 45)	*Q*3 (n = 45)	*Q*4 (n = 45)	Std. diff.*
Age, yr, mean ± SD	60.72 ± 8.91 (n = 45)	60.15 ± 9.03 (n = 45)	59.88 ± 8.67 (n = 45)	60.31 ± 8.42 (n = 45)	.04
Male sex, n/N (%)	26/45 (57.8)	24/45 (53.3)	25/45 (55.6)	23/45 (51.1)	.09
Education ≤ high school, n/N (%)	22/45 (48.9)	24/45 (53.3)	23/45 (51.1)	21/45 (46.7)	.08
Married, n/N (%)	34/45 (75.6)	33/45 (73.3)	35/45 (77.8)	34/45 (75.6)	.05
Monthly income < 5000, n/N (%)	18/45 (40.0)	20/45 (44.4)	19/45 (42.2)	17/45 (37.8)	.07
Cancer type, n/N (%)					.10
Gastrointestinal	17/45 (37.8)	18/45 (40.0)	16/45 (35.6)	17/45 (37.8)	
Lung	11/45 (24.4)	10/45 (22.2)	12/45 (26.7)	11/45 (24.4)	
Breast	7/45 (15.6)	8/45 (17.8)	7/45 (15.6)	8/45 (17.8)	
Other solid tumors	10/45 (22.2)	9/45 (20.0)	10/45 (22.2)	9/45 (20.0)	
Stage IV disease, n/N (%)	31/45 (68.9)	32/45 (71.1)	33/45 (73.3)	34/45 (75.6)	.12
≥2 metastatic sites, n/N (%)	20/45 (44.4)	22/45 (48.9)	24/45 (53.3)	26/45 (57.8)	.18
ECOG PS 0–1, n/N (%)	38/45 (84.4)	37/45 (82.2)	36/45 (80.0)	34/45 (75.6)	.21
≥2 comorbidities, n/N (%)	19/45 (42.2)	18/45 (40.0)	20/45 (44.4)	21/45 (46.7)	.09
Global QOL score, mean ± SD	55.23 ± 17.34 (n = 45)	54.87 ± 16.92 (n = 45)	53.95 ± 17.10 (n = 45)	54.66 ± 16.83 (n = 45)	.07
ESAS total score, mean ± SD	37.45 ± 15.28 (n = 45)	38.12 ± 14.97 (n = 45)	39.03 ± 15.64 (n = 45)	39.88 ± 15.21 (n = 45)	.15
Coping score (Brief COPE), mean ± SD	62.13 ± 11.25 (n = 45)	62.54 ± 10.98 (n = 45)	61.87 ± 11.43 (n = 45)	62.01 ± 11.10 (n = 45)	.05
Self-management capability (SMC), mean ± SD	58.21 ± 12.36 (n = 45)	58.92 ± 12.10 (n = 45)	59.34 ± 11.98 (n = 45)	59.87 ± 11.76 (n = 45)	.14

Data presentation: Continuous variables are presented as mean ± standard deviation; categorical variables as n/N (%), with denominators reflecting non-missing observations for each variable.

Missing data: No missing data were observed for baseline variables shown in this table unless otherwise indicated.

Standardized differences: Std. Diff. represents the maximum standardized difference across SCI quartiles; values < 0.10 indicate good balance, and values < 0.20 indicate acceptable balance.

Scope of SCI: SCI reflects nurse-delivered supportive care encounters only and does not include supportive care services delivered outside nursing (e.g., nutrition counseling, psychological services, or palliative care consultations).

ECOG = Eastern Cooperative Oncology Group, ESAS = Edmonton Symptom Assessment System, QOL = quality of life, SCI, supportive care intensity, SD = standard deviation, SMC = self-management capability.

Sociodemographic characteristics showed small standardized differences across SCI quartiles. The mean age ranged from 59.88 to 60.72 years, and the proportion of male patients ranged from 51.11% to 57.78%. Education level, marital status, and monthly income were also generally similar across groups.

Clinical characteristics were largely similar across SCI quartiles, but selected disease-related variables showed modest imbalance. The prevalence of stage IV disease increased from 68.89% in *Q*1 to 75.56% in *Q*4, and the proportion of patients with ≥2 metastatic sites increased from 44.44% to 57.78%. ECOG performance status showed the largest standardized difference, with the proportion of patients classified as ECOG PS 0 to 1 decreasing from 84.44% in *Q*1 to 75.56% in *Q*4 and a standardized difference of 0.21. This modest imbalance was considered in subsequent adjusted analyses and in the interpretation of potential confounding by baseline clinical need.

Baseline patient-reported outcomes were generally similar across SCI quartiles. Global QOL scores ranged from 53.95 to 55.23, ESAS total scores ranged from 37.45 to 39.88, Brief COPE scores ranged from 61.87 to 62.54, and SMC scores ranged from 58.21 to 59.87. These baseline patient-reported measures were adjusted for, where appropriate, in the main longitudinal and survival models.

### 3.2. Supportive care intensity

SCI scores showed substantial variability across the cohort, ranging from 2 to 13 with a median of 8.00 (IQR: 6.00–10.00) (Table 2). Quartile grouping demonstrated clear separation of SCI levels. Higher SCI quartiles corresponded to progressively greater engagement in supportive care activities, including structured symptom assessments, evidence-based symptom education, coping-enhancement counseling, self-management coaching, caregiver involvement, and telephone follow-up contacts (all *P* < .001; Table S1, Supplemental Digital Content 1). Logistic regression indicated that patients with higher baseline symptom burden (adjusted OR = 1.21, 95% CI 1.04–1.41) and lower baseline global QOL (adjusted OR = 1.19, 95% CI 1.01–1.41) were more likely to receive high-intensity supportive care, as were those with caregiver involvement (adjusted OR = 1.89, 95% CI 1.06–3.38) (Table S2, Supplemental Digital Content 2).

**Table 2:** Distribution of supportive care intensity (SCI) scores

Variable	Mean ± SD	Median (IQR)	Range
Overall (N = 180)	7.84 ± 2.61	8.00 (6.00–10.00)	2–13
*Q*1 (n = 45)	4.12 ± 0.83	4.00 (3.50–4.50)	2–5
*Q*2 (n = 45)	6.35 ± 0.49	6.00 (6.00–7.00)	6–7
*Q*3 (n = 45)	9.02 ± 0.73	9.00 (8.50–9.50)	8–10
*Q*4 (n = 45)	12.01 ± 0.78	12.00 (11.50–12.50)	11–13

Data presentation: Continuous variables are presented as mean ± standard deviation and median (interquartile range).

Missing data: No missing data were observed for SCI score calculation (N = 180 for all categories).

Scope of SCI: SCI reflects nurse-delivered supportive care encounters only and does not include supportive care services delivered outside nursing (e.g., nutrition counseling, psychological services, or palliative care consultations).

IQR = interquartile range, SCI = supportive care intensity, SD = standard deviation.

### 3.3. Primary outcome

Global QOL improved over time, with the greatest gains observed at 18 weeks. In the mixed-effects model (Table 3), higher supportive care intensity was independently associated with better global QOL (*P* < .001), and a significant SCI × time interaction indicated a dose–response pattern, whereby patients receiving higher-intensity care experienced larger improvements.

**Table 3:** Longitudinal mixed-effects model for global quality of life (EORTC QLQ-C30).

Variable	β (95% CI)	*P* value
Time (vs baseline)		
12 wk	3.42 (0.88–5.96)	.009
18 wk	6.87 (3.21–10.42)	<.001
24 wk	4.11 (0.55–7.66)	.024
Supportive care intensity (per quartile increase)	2.84 (1.32–4.36)	<.001
SCI × time interaction		
12 wk × SCI	1.12 (0.10–2.14)	.032
18 wk × SCI	2.01 (0.99–3.03)	<.001
24 wk × SCI	0.98 (−0.08 to 2.03)	.071
Covariates		
Age (per 10 yr)	−0.84 (−2.13 to 0.46)	.205
Female sex	1.78 (−0.95 to 4.51)	.196
ECOG performance status ≥ 2	−4.33 (−7.88 to − 0.78)	.017
Baseline ESAS (per 5-point increase)	−1.92 (−3.40 to − 0.44)	.011

Model specification: Estimates are derived from linear mixed-effects models with participant-level random intercepts and an unstructured covariance matrix to account for within-subject correlation across follow-up time points.

Missing data and attrition: All available observations were included under a missing-at-random assumption inherent in mixed-effects modeling; no imputation was applied for longitudinal outcomes.

Exposure definition: Supportive care intensity (SCI) was modeled as a cumulative exposure (per quartile increase) and not as a time-varying covariate; potential time-varying confounding due to escalation of supportive care in response to worsening symptoms was not explicitly modeled.

Adjustment variables: Models were adjusted for age, sex, ECOG performance status, and baseline ESAS score.

Interpretation: Higher β coefficients indicate higher global quality-of-life scores. Reported estimates reflect average longitudinal associations over follow-up rather than time-specific causal effects.

CI = confidence interval, ECOG = Eastern Cooperative Oncology Group, EORTC = European Organization for Research and Treatment of Cancer, ESAS = Edmonton Symptom Assessment System, SCI = supportive care intensity.

Across QOL subdomains, physical and role functioning showed the largest benefits, and fatigue, appetite loss, and constipation improved significantly with higher SCI exposure (Table S3, Supplemental Digital Content 3). In addition, patients receiving higher supportive care intensity were more likely to achieve a clinically meaningful QOL improvement of ≥10 points (adjusted OR 1.46, 95% CI 1.12–1.90) after adjustment for baseline status and covariates (Table S4, Supplemental Digital Content 4).

### 3.4. Symptom burden, coping, self-management, and existential well-being

Symptom burden declined over time, with significantly larger reductions in patients receiving higher supportive care intensity. In the mixed-effects model (Table 4), higher SCI was associated with lower ESAS total scores (β − 2.21, *P* < .001) and stronger improvements at 18 and 24 weeks. Symptom-specific analyses (Table S5, Supplemental Digital Content 5) showed the greatest benefits in fatigue, appetite loss, sleep disturbance, and overall well-being. Despite this, 24.4% remained highly symptomatic at 24 weeks. Persistent high ESAS (≥30) was predicted by higher baseline ESAS, ECOG ≥ 2, and baseline anxiety/depression, whereas higher SCI was protective (adjusted OR 0.71, *P* = .007; Table S6, Supplemental Digital Content 6).

**Table 4:** Longitudinal mixed-effects model for ESAS total symptom burden.

Variable	β (95% CI)	*P* value
Time (vs baseline)		
12 wk	−3.12 (−5.01 to − 1.22)	.001
18 wk	−5.46 (−7.82 to − 3.10)	<.001
24 wk	−4.08 (−6.61 to − 1.55)	.002
Supportive care intensity (per quartile increase)	−2.21 (−3.41 to − 1.01)	<.001
SCI × Time interaction		
12 wk × SCI	−0.88 (−1.60 to − 0.16)	.017
18 wk × SCI	−1.47 (−2.24 to − 0.69)	<.001
24 wk × SCI	−0.92 (−1.72 to − 0.12)	.025
Covariates		
Age (per 10 yr)	0.31 (−0.41–1.03)	.394
Female sex	1.24 (−0.88–3.36)	.252
ECOG performance status ≥ 2	4.51 (1.93–7.09)	.001
Baseline QOL (per 10-point decrease)	1.02 (0.12–1.92)	.027

Model specification: Estimates are derived from linear mixed-effects models with participant-level random intercepts and an unstructured covariance matrix to account for within-subject correlation across follow-up time points.

Missing data and attrition: All available observations were included under a missing-at-random assumption inherent in mixed-effects modeling; no imputation was applied for longitudinal outcomes.

Exposure definition: Supportive care intensity (SCI) was modeled as a cumulative exposure (per quartile increase) and not as a time-varying covariate; potential time-varying confounding due to escalation of supportive care in response to worsening symptoms was not explicitly modeled.

Adjustment variables: Models were adjusted for age, sex, ECOG performance status, and baseline global quality-of-life score.

Interpretation: Negative β coefficients indicate lower ESAS total scores (reduced symptom burden). Reported estimates reflect average longitudinal associations over follow-up rather than time-specific causal effects.

CI = confidence interval, ECOG = Eastern Cooperative Oncology Group, ESAS = Edmonton Symptom Assessment System, QOL= quality of life, SCI = supportive care intensity.

Significant gains were observed in active coping, positive reframing, acceptance, and emotional support use by 18 weeks (all *P* < .01), while maladaptive strategies declined (Table S7, Supplemental Digital Content 7). Higher SCI was a strong predictor of achieving high SMC at 24 weeks (adjusted OR 1.82, *P* < .001), along with baseline SMC and caregiver involvement (Table S8, Supplemental Digital Content 8). EB improved modestly over 24 weeks, with higher SCI independently associated with better EB outcomes (β 1.28, *P* = .004). Lower baseline EB and ECOG ≥ 2 predicted poorer 24-week EB (Table S9, Supplemental Digital Content 9).

### 3.5. Subgroup and sensitivity analyses

Subgroup analyses demonstrated that the associations between higher supportive care intensity and improvements in global QOL and ESAS were directionally consistent across age, sex, ECOG performance status, and cancer type. No significant interaction effects were observed (all *P*-interaction >.05), indicating stable effects across subgroups (Table S10, Supplemental Digital Content 10).

Results remained robust across all sensitivity analyses. Effect estimates were nearly identical in complete-case versus multiple-imputation models (Table S11, Supplemental Digital Content 11). Excluding participants with early death or substantial dropout also did not materially alter the associations for either QOL or ESAS (Table S12, Supplemental Digital Content 12). Across all sensitivity checks, higher SCI continued to predict greater QOL improvement and lower symptom burden.

### 3.6. Survival analysis

A total of 180 patients were included in the survival analysis. During a median follow-up of 14.8 months (IQR: 9.2–21.3), 92 patients (51.1%) died, and 18 patients (10.0%) were lost to follow-up and censored at their last known contact. Follow-up completeness exceeded 90% at 12 months and remained above 80% at 24 months, with comparable event distributions across supportive care intensity groups (Table S13, Supplemental Digital Content 13).

**Figure 1.**
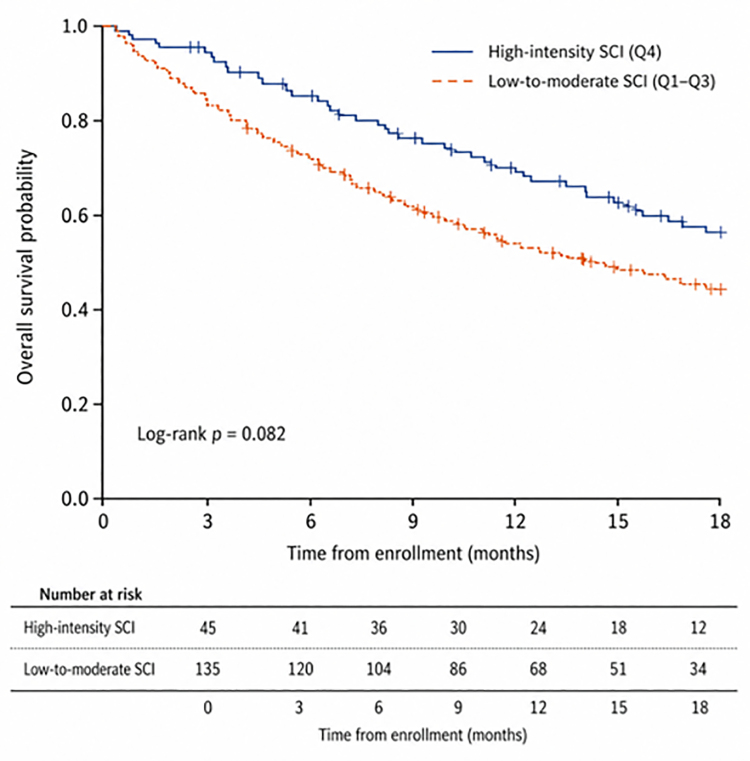
Kaplan–Meier curves for overall survival by supportive care intensity. Kaplan–Meier estimates of overall survival among patients receiving high versus low-to-moderate nurse-led supportive care intensity. High-intensity care was defined as the highest quartile of the supportive care intensity (SCI) score (*Q*4), whereas low-to-moderate intensity included quartiles *Q*1 to *Q*3. Survival probabilities were compared using the log-rank test. Shaded areas indicate 95% confidence intervals. Numbers at risk are shown below the *x*-axis, and censoring is indicated by tick marks.

Kaplan–Meier curves demonstrated a consistent separation between the high-intensity and low-to-moderate supportive care groups (Fig. 1). Patients receiving high-intensity nurse-led supportive care showed numerically higher survival probabilities at 12 and 24 months, although the difference did not reach statistical significance in the log-rank test (*P* = .082).

In multivariable Cox proportional hazards models (Table 5), higher supportive care intensity was independently associated with a reduced risk of all-cause mortality (adjusted HR 0.68; 95% CI 0.48–0.96; *P* = .030), after adjustment for age, sex, cancer type, ECOG performance status, baseline ESAS total score, and baseline global QOL. Sensitivity analyses using complete-case data and multiple imputation produced consistent effect estimates (Table S14, Supplemental Digital Content 14).

**Table 5:** Multivariable Cox proportional hazards models for overall survival.

Variable	Adjusted HR (95% CI)	*P* value
Supportive care intensity (per + 1 SCI)	0.88 (0.79–0.98)	.021
High SCI (*Q*4 vs *Q*1–*Q*3)	0.68 (0.48–0.96)	.030
Age (per 10-yr increase)	1.12 (0.94–1.34)	.210
Male sex	1.18 (0.78–1.80)	.430
ECOG performance status ≥ 2	1.96 (1.29–2.98)	.001
Cancer type: gastrointestinal vs others	1.24 (0.83–1.86)	.290
Baseline global QOL (per 10-point increase)	0.84 (0.73–0.96)	.012
Baseline ESAS (per 5-point increase)	1.17 (1.03–1.33)	.014

Model adjustment: Models were adjusted for age, sex, ECOG performance status, cancer type, baseline global quality of life, and baseline symptom burden.

Proportional hazards assumption: Assessed using Schoenfeld residuals; no material violations were observed.

Exposure definition: Supportive care intensity (SCI) was treated as a cumulative exposure and not as a time-varying covariate.

Mediation interpretation: No prespecified causal mediation framework with defined temporal ordering was estimated. Any mediation-related interpretations are exploratory and descriptive and should not be interpreted as evidence of causal indirect effects.

Interpretation: HR < 1 indicates a reduced risk of mortality associated with higher supportive care intensity.

CI = confidence interval, ECOG = Eastern Cooperative Oncology Group, ESAS = Edmonton Symptom Assessment System, HR = hazard ratio, QOL= quality of life, SCI = supportive care intensity.

Exploratory dose–response analyses further suggested a possible contact-frequency pattern, whereby patients receiving ≥8 nurse-led supportive-care contacts had lower mortality risk than those receiving fewer contacts, with an adjusted HR of 0.61 (95% CI 0.40–0.92; *P* = .018) (Table S15, Supplemental Digital Content 15). Exploratory pathway analyses further suggested that higher supportive care intensity was associated with early improvements in global QOL and SMC, both of which were in turn associated with lower subsequent mortality risk (Table S16, Supplemental Digital Content 16). These exploratory associations are descriptive and should not be interpreted as evidence of indirect effects or causal mediation.

## 4. Discussion

This study quantified nurse-led supportive care intensity and examined its associations with multiple patient outcomes in a real-world palliative chemotherapy setting. Higher supportive care intensity was associated with greater improvements in global QOL between 18 and 24 weeks, better physical and role functioning, and larger reductions in fatigue, appetite loss, constipation, and overall symptom burden. Patients receiving higher-intensity care also demonstrated more adaptive coping strategies, gains across domains of SMC, and better existential well-being. In exploratory survival analyses, higher SCI was associated with a lower adjusted risk of mortality in multivariable Cox models, with a possible contact-frequency pattern observed among patients receiving 8 or more nursing encounters. Taken together, these findings suggest that higher nurse-led supportive care intensity was associated with better QOL, symptom, coping, and self-management outcomes, whereas the survival findings should be interpreted cautiously because of the observational design and potential residual and time-varying confounding.

Our findings are generally consistent with evidence from early palliative care trials, which have reported improvements in QOL, reductions in symptom burden, and gains in coping and self-efficacy – often emerging within the 1st 12 to 18 weeks of care.^[20,24,26–28]^ In line with these studies, higher supportive care intensity in our cohort was associated with multidimensional patient-reported improvements.^[29,30]^ However, an important distinction of this study is its nurse-led, non-protocolized approach, which reflects routine clinical practice more closely than highly structured RCT interventions. This real-world design suggests that clinically relevant patient-reported improvements may be achievable through flexible, nurse-driven supportive care processes, while causal interpretation remains limited by the observational design.^[31,32]^

The greater reductions in symptom burden observed among patients receiving higher supportive care intensity may be explained by several clinically plausible mechanisms. More frequent nursing contact may facilitate earlier detection of emerging symptoms and more timely responses, potentially limiting symptom escalation and reducing overall distress.^[33,34]^ In addition, structured symptom education may help patients develop practical strategies for managing pain, nausea, fatigue, and other common treatment-related symptoms.^[35]^ Enhanced understanding and skill acquisition may strengthen patients’ perceived control over symptoms, which could contribute to lower distress and a more manageable symptom experience.^[36,37]^

The observed enhancements in coping and SMC are consistent with theoretical models underlying nurse-delivered psychosocial support.^[18,27,28]^ Components of supportive care may overlap with principles of Acceptance and Commitment Therapy, which encourages patients to acknowledge symptoms without overreacting to them and to engage in value-guided actions that support adaptive functioning.^[38–42]^ Similarly, principles from social cognitive theory may help explain how repeated nursing support could strengthen self-efficacy and patients’ confidence in managing symptoms and treatment demands.^[39–41]^ Together, these frameworks provide a plausible explanation for why higher nurse-led supportive care intensity was associated with more adaptive coping patterns and greater perceived SMC.

The favorable survival association observed among patients receiving higher supportive care intensity should be interpreted cautiously. Improved QOL, lower symptom burden, and greater SMC are clinically plausible factors that may help explain the observed association, but the present study did not estimate formal causal pathways or indirect effects. More frequent nursing contact may also reflect closer clinical surveillance and more timely recognition of complications, which could be relevant to subsequent outcomes.^[2,43–46]^ However, these explanations remain hypothesis-generating because residual confounding, differential caregiver support, and time-varying allocation of supportive care cannot be excluded.

The observed associations between higher supportive care intensity and improvements in symptom burden, coping, SMC, and existential well-being suggest clinically plausible explanatory links with downstream QOL trajectories and survival-related outcomes. However, these pathway-related observations should be interpreted as conceptual and exploratory rather than as evidence of causal mechanisms. In the present study, we did not specify a formal mediation model, define individual-level temporal ordering between exposure, intermediate outcomes, and survival, or invoke assumptions required for causal indirect-effect estimation. Accordingly, these exploratory pathway-related analyses should be viewed as descriptive associations intended to inform future hypothesis testing, rather than as estimates of direct or indirect causal effects.

The exploratory contact-frequency findings should be interpreted as context-specific rather than as evidence of a universal intensity threshold. Patients receiving 8 or more nurse-led supportive care encounters had more favorable adjusted survival estimates, but this observation requires validation in larger multicenter cohorts. Encounter frequency may nevertheless represent a pragmatic process measure that is feasible to document in routine oncology nursing practice and may inform future implementation studies. Collectively, these findings highlight the central role of nurses in supportive oncology care and support further evaluation of structured nurse-led supportive care processes within routine chemotherapy practice. Digital tools, such as remote symptom monitoring, may further enhance the reach and continuity of nurse-led support.

Although supportive care intensity was associated with clinically relevant patient-reported outcomes in this study, it is not intended as a benchmarking metric for cross-institutional comparison in its current form. SCI should instead be viewed as a process-oriented measure describing nursing supportive care delivery within a given care context. For reproducibility, calculation of SCI requires a minimum set of standardized data elements, including documentation of nurse–patient supportive care encounters (frequency and mode), encounter duration, and prespecified supportive care content domains such as symptom assessment, education, coping support, self-management coaching, and caregiver engagement. Any future use of SCI for benchmarking would additionally require robust case-mix adjustment using variables such as cancer type, disease stage, performance status, and baseline symptom burden, as well as external validation across diverse oncology settings. Thresholds identified in the present study should therefore be interpreted as exploratory and context-specific rather than as universal performance standards.

Several sources of bias warrant consideration when interpreting these findings. First, confounding by baseline need is likely, as patients with greater symptom burden, poorer baseline QOL, or worse performance status may have been more likely to receive higher-intensity nurse-led supportive care. Although we adjusted for baseline symptom severity, functional status, disease characteristics, and other key clinical and demographic characteristics based on a prespecified conceptual framework, residual confounding cannot be fully excluded. Such confounding by indication may have attenuated favorable associations if sicker patients preferentially received more intensive nursing support; however, other unmeasured factors could have biased estimates in the opposite direction.

In addition, differential access to informal caregivers or family support – factors not comprehensively captured in our data – could bias associations in either direction, depending on whether caregiver availability facilitated both higher supportive care intensity and better outcomes. Other plausible unmeasured confounders include health literacy, patient engagement, clinician referral patterns, and institutional workflow factors, all of which may influence both receipt of nurse-led supportive care and patient-reported outcomes. These considerations underscore the need for cautious causal interpretation and highlight the importance of future studies with more granular measurement of social and care-contextual factors.

Several methodological limitations should also be acknowledged. Although SCI components were prespecified and documentation was standardized through structured nurse training, standardized templates, and routine supervisory review, formal prospective inter-rater reliability testing was not performed. Measurement error in SCI classification therefore cannot be excluded and may have attenuated the observed associations. Supportive care intensity may also have increased in response to worsening symptoms during follow-up, introducing time-varying confounding that was not explicitly addressed using causal methods. This could have biased estimates in either direction, although escalation of care in response to worsening symptoms may plausibly have attenuated favorable associations. The study was conducted at a single center, which may limit generalizability, and the number of survival events was modest, necessitating cautious interpretation of exploratory survival findings. Additional limitations include reliance on self-reported coping and self-management measures, absence of objective biomarkers such as inflammatory or nutritional indices, lack of blinding, and approximately 20% attrition in longitudinal patient-reported outcome assessments, which may have affected precision. Given the observational design, causal inference cannot be established. Future multicenter studies with larger samples should incorporate formal reliability assessment, more granular measurement of social and care-contextual factors, and analytic approaches capable of addressing time-varying confounding.

## 5. Conclusion

This study shows that higher intensity of nurse-led supportive care was associated with meaningful improvements in QOL, symptom burden, coping, and SMC among patients with advanced cancer, and with a favorable exploratory association with lower adjusted mortality risk. These findings underscore the central role of nurses in palliative and supportive oncology care, while causal interpretation should remain cautious because of the observational design and potential residual and time-varying confounding. From a pragmatic perspective, achieving higher supportive care intensity may be feasible through structured prioritization of supportive care encounters within existing nursing workflows and the use of scalable approaches such as proactive follow-up and remote symptom monitoring, rather than expansion of specialized services alone. Supportive care intensity should therefore be viewed as a process-oriented indicator of nursing practice patterns, with implementation tailored to local staffing models, patient case mix, and care contexts.

## Acknowledgments

The authors sincerely thank all study participants for their invaluable contributions.

## Author contributions

**Conceptualization:** Bao-Mei Wang, Wen-Ling Wang, Yong-Jian Zhang, Meng Wu, Hai-Xia Wei, Shu-Yan Zhang, Meng Li, Bo-Bo Yan, Wen Zhang.

**Data curation:** Wen Zhang.

**Methodology:** Wen Zhang.

**Resources:** Wen Zhang.

**Software:** Wen Zhang.

**Supervision:** Wen Zhang.

**Validation:** Wen Zhang.

**Visualization:** Wen Zhang.

**Writing – original draft:** Wen Zhang.

**Writing – review & editing:** Wen Zhang.
































