# Supplementary material for: Impact of nurse-led supportive care intensity on quality of life and symptom burden in patients undergoing palliative chemotherapy: A prospective cohort study
Source: Medicine (Baltimore). 2026 Jul 24;105(30):e49780. doi: 10.1097/MD.0000000000049780 (PMC13406126; doi:10.1097/MD.0000000000049780)
Supplement: Supplementary file 1 [file medi-105-e49780-s001.docx]

**Supplementary Table S1. Components of Nurse-Led Supportive Care Intensity by Quartiles**

| **Component** | **Q1 (n=45)** | **Q2 (n=45)** | **Q3 (n=45)** | **Q4 (n=45)** | **p-value** |
| --- | --- | --- | --- | --- | --- |
| Structured symptom assessments ≥1× per cycle, n (%) | 12 (26.67) | 24 (53.33) | 36 (80.00) | 42 (93.33) | <0.001 |
| Symptom education (EB-based), n (%) | 10 (22.22) | 22 (48.89) | 34 (75.56) | 40 (88.89) | <0.001 |
| Coping-enhancement counseling (≥3 sessions), n (%) | 5 (11.11) | 14 (31.11) | 28 (62.22) | 35 (77.78) | <0.001 |
| Self-management coaching, n (%) | 8 (17.78) | 20 (44.44) | 33 (73.33) | 39 (86.67) | <0.001 |
| Caregiver involvement, n (%) | 7 (15.56) | 16 (35.56) | 27 (60.00) | 32 (71.11) | <0.001 |
| Telephone follow-ups, mean ± SD | 0.78 ± 0.96 | 2.11 ± 1.32 | 3.84 ± 1.75 | 5.63 ± 1.98 | <0.001 |
| Total supportive-care encounters, mean ± SD | 2.21 ± 1.03 | 4.02 ± 1.41 | 6.48 ± 2.03 | 8.92 ± 2.37 | <0.001 |

**Footnotes:**
Percentages presented to two decimals.
P-values derived from χ² tests (categorical variables) or ANOVA/Kruskal–Wallis tests (continuous variables).
