# Supplementary material for: Impact of nurse-led supportive care intensity on quality of life and symptom burden in patients undergoing palliative chemotherapy: A prospective cohort study
Source: Medicine (Baltimore). 2026 Jul 24;105(30):e49780. doi: 10.1097/MD.0000000000049780 (PMC13406126; doi:10.1097/MD.0000000000049780)
Supplement: Supplementary file 2 [file medi-105-e49780-s002.docx]

**Supplementary Table S2. Predictors of High-Intensity Supportive Care (Logistic Regression)**

| **Variable** | **Crude OR (95% CI)** | **p-value** | **Adjusted ORa (95% CI)** | **p-value** |
| --- | --- | --- | --- | --- |
| Age (per 10-year increase) | 0.94 (0.76–1.16) | 0.565 | 0.91 (0.72–1.15) | 0.423 |
| Male sex | 0.88 (0.52–1.49) | 0.629 | 0.84 (0.47–1.50) | 0.558 |
| Education > high school | 1.32 (0.77–2.27) | 0.309 | 1.41 (0.79–2.52) | 0.242 |
| ECOG 0–1 | 1.56 (0.81–3.01) | 0.184 | 1.72 (0.86–3.46) | 0.123 |
| Baseline ESAS (per 5-point increase) | 1.18 (1.02–1.37) | 0.027 | 1.21 (1.04–1.41) | 0.016 |
| Baseline global QOL (per 10-point decrease) | 1.15 (0.98–1.35) | 0.084 | 1.19 (1.01–1.41) | 0.039 |
| Caregiver involvement | 1.74 (1.02–2.98) | 0.041 | 1.89 (1.06–3.38) | 0.031 |

**Footnotes:**
High-intensity supportive care defined as SCI quartile Q4.
a Adjusted for age, sex, education, ECOG status, cancer type, baseline ESAS, baseline global QOL.
