# Supplementary material for: Impact of nurse-led supportive care intensity on quality of life and symptom burden in patients undergoing palliative chemotherapy: A prospective cohort study
Source: Medicine (Baltimore). 2026 Jul 24;105(30):e49780. doi: 10.1097/MD.0000000000049780 (PMC13406126; doi:10.1097/MD.0000000000049780)
Supplement: Supplementary file 3 [file medi-105-e49780-s003.docx]

**Supplementary Table S3. Effects of Supportive Care Intensity on QOL Subdomains at 18 Weeks**

| **QOL Subdomain** | **β (95% CI)** | **p-value** |
| --- | --- | --- |
| Physical functioning | 5.21 (2.10 to 8.32) | 0.001 |
| Role functioning | 6.48 (3.05 to 9.91) | <0.001 |
| Emotional functioning | 2.01 (−0.84 to 4.86) | 0.163 |
| Cognitive functioning | 1.54 (−0.62 to 3.70) | 0.160 |
| Social functioning | 2.78 (−0.40 to 5.95) | 0.086 |
| Fatigue (−) | −6.42 (−9.87 to −2.98) | <0.001 |
| Pain (−) | −3.15 (−6.22 to −0.09) | 0.044 |
| Nausea/Vomiting (−) | −2.22 (−4.88 to 0.43) | 0.101 |
| Appetite loss (−) | −5.91 (−9.74 to −2.09) | 0.002 |
| Constipation (−) | −4.53 (−7.82 to −1.24) | 0.007 |

**Footnote:**
Negative β indicates reduced symptom burden (improvement). Adjusted for age, sex, ECOG status, and baseline ESAS.
