# Supplementary material for: Impact of nurse-led supportive care intensity on quality of life and symptom burden in patients undergoing palliative chemotherapy: A prospective cohort study
Source: Medicine (Baltimore). 2026 Jul 24;105(30):e49780. doi: 10.1097/MD.0000000000049780 (PMC13406126; doi:10.1097/MD.0000000000049780)
Supplement: Supplementary file 4 [file medi-105-e49780-s004.docx]

**Supplementary Table S4. Predictors of Clinically Meaningful QOL Improvement (≥10-Point Increase)**

| **Variable** | **Adjusted OR (95% CI)** | **p-value** |
| --- | --- | --- |
| Supportive Care Intensity (per quartile) | 1.46 (1.12–1.90) | 0.005 |
| Age (per 10 years) | 0.94 (0.73–1.22) | 0.657 |
| Female sex | 1.18 (0.68–2.04) | 0.559 |
| ECOG 0–1 | 1.72 (1.01–3.14) | 0.046 |
| Baseline QOL (per 10-point decrease) | 1.28 (1.06–1.55) | 0.010 |
| Baseline ESAS (per 5 points) | 1.24 (1.03–1.49) | 0.021 |
| Cancer type (GI vs others) | 0.91 (0.48–1.76) | 0.785 |
| Caregiver involvement | 1.67 (0.98–2.87) | 0.061 |

**Footnote:**
Outcome = ≥10-point improvement in global QOL from baseline. Adjusted for age, sex, ECOG, cancer type, baseline QOL, and baseline ESAS.
