# Supplementary material for: Impact of nurse-led supportive care intensity on quality of life and symptom burden in patients undergoing palliative chemotherapy: A prospective cohort study
Source: Medicine (Baltimore). 2026 Jul 24;105(30):e49780. doi: 10.1097/MD.0000000000049780 (PMC13406126; doi:10.1097/MD.0000000000049780)
Supplement: Supplementary file 5 [file medi-105-e49780-s005.docx]

**Supplementary Table S5. Symptom-Specific ESAS Changes Associated With SCI (18 Weeks)**

| **ESAS Symptom** | **β (95% CI)** | **p-value** |
| --- | --- | --- |
| Pain | −2.14 (−3.51 to −0.77) | 0.002 |
| Fatigue | −3.85 (−5.42 to −2.29) | <0.001 |
| Drowsiness | −1.72 (−2.93 to −0.51) | 0.006 |
| Nausea | −0.88 (−1.92 to 0.16) | 0.097 |
| Appetite loss | −3.31 (−5.01 to −1.61) | <0.001 |
| Dyspnea | −1.46 (−2.64 to −0.28) | 0.016 |
| Depression | −1.21 (−2.44 to 0.02) | 0.054 |
| Anxiety | −1.65 (−2.89 to −0.41) | 0.009 |
| Sleep disturbance | −2.18 (−3.61 to −0.75) | 0.003 |
| Well-being (reverse-coded) | −2.92 (−4.33 to −1.51) | <0.001 |
