# Supplementary material for: Impact of nurse-led supportive care intensity on quality of life and symptom burden in patients undergoing palliative chemotherapy: A prospective cohort study
Source: Medicine (Baltimore). 2026 Jul 24;105(30):e49780. doi: 10.1097/MD.0000000000049780 (PMC13406126; doi:10.1097/MD.0000000000049780)
Supplement: Supplementary file 6 [file medi-105-e49780-s006.docx]

**Supplementary Table S6. Logistic Regression for Persistent High Symptom Burden at 24 Weeks (ESAS ≥ 30)**

| **Variable** | **Adjusted OR (95% CI)** | **p-value** |
| --- | --- | --- |
| Supportive Care Intensity (per quartile) | 0.71 (0.55–0.91) | 0.007 |
| Baseline ESAS (per 5 points) | 1.38 (1.14–1.67) | 0.001 |
| ECOG ≥2 | 2.31 (1.18–4.42) | 0.014 |
| Cancer type (GI vs others) | 1.22 (0.65–2.30) | 0.534 |
| Baseline HADS-Anxiety | 1.09 (1.02–1.17) | 0.013 |
| Baseline HADS-Depression | 1.11 (1.04–1.19) | 0.003 |
| Caregiver involvement | 0.84 (0.48–1.49) | 0.561 |
| Age (per 10 years) | 1.03 (0.82–1.30) | 0.784 |
