# Supplementary material for: Impact of nurse-led supportive care intensity on quality of life and symptom burden in patients undergoing palliative chemotherapy: A prospective cohort study
Source: Medicine (Baltimore). 2026 Jul 24;105(30):e49780. doi: 10.1097/MD.0000000000049780 (PMC13406126; doi:10.1097/MD.0000000000049780)
Supplement: Supplementary file 7 [file medi-105-e49780-s007.docx]

**Supplementary Table S7. Longitudinal Mixed-Effects Model for Coping (Brief COPE)**

| **Coping Domain** | **β at 18 Weeks (95% CI)** | **p-value** |
| --- | --- | --- |
| Active coping | 2.48 (1.22 to 3.74) | <0.001 |
| Positive reframing | 1.97 (0.88 to 3.06) | 0.001 |
| Acceptance | 1.52 (0.51 to 2.52) | 0.003 |
| Emotional support use | 1.24 (0.19 to 2.28) | 0.021 |
| Instrumental support | 0.88 (−0.21 to 1.96) | 0.113 |
| Behavioral disengagement (−) | −0.92 (−1.78 to −0.06) | 0.037 |
| Self-blame (−) | −1.28 (−2.33 to −0.23) | 0.017 |

**Footnote:** Positive β = improvement in adaptive coping; negative β = reduction in maladaptive coping.
