# Supplementary material for: Impact of nurse-led supportive care intensity on quality of life and symptom burden in patients undergoing palliative chemotherapy: A prospective cohort study
Source: Medicine (Baltimore). 2026 Jul 24;105(30):e49780. doi: 10.1097/MD.0000000000049780 (PMC13406126; doi:10.1097/MD.0000000000049780)
Supplement: Supplementary file 8 [file medi-105-e49780-s008.docx]

**Supplementary Table S8. Logistic Regression for High Self-Management Capability at 24 Weeks (≥P75)**

| **Variable** | **Adjusted OR (95% CI)** | **p-value** |
| --- | --- | --- |
| Supportive Care Intensity (per quartile) | 1.82 (1.32–2.53) | <0.001 |
| Baseline SMC (per 5 points) | 1.41 (1.19–1.70) | <0.001 |
| Education (college or above) | 1.56 (0.88–2.77) | 0.127 |
| Caregiver involvement | 1.91 (1.10–3.35) | 0.021 |
| Age (per 10 years) | 0.89 (0.68–1.17) | 0.402 |
| Female sex | 1.12 (0.65–1.93) | 0.688 |
