# Supplementary material for: Impact of nurse-led supportive care intensity on quality of life and symptom burden in patients undergoing palliative chemotherapy: A prospective cohort study
Source: Medicine (Baltimore). 2026 Jul 24;105(30):e49780. doi: 10.1097/MD.0000000000049780 (PMC13406126; doi:10.1097/MD.0000000000049780)
Supplement: Supplementary file 9 [file medi-105-e49780-s009.docx]

**Supplementary Table S9. Existential Well-Being at 24 Weeks (FACIT-Sp Subscale)**

| **Variable** | **β (95% CI)** | **p-value** |
| --- | --- | --- |
| Supportive Care Intensity (per quartile) | 1.28 (0.41 to 2.15) | 0.004 |
| Baseline EB score | −0.34 (−0.52 to −0.16) | <0.001 |
| Age (per 10 years) | 0.22 (−0.19 to 0.62) | 0.294 |
| Female sex | 0.88 (−0.73 to 2.48) | 0.282 |
| ECOG ≥2 | −1.64 (−3.09 to −0.19) | 0.027 |
