# Supplementary material for: Impact of nurse-led supportive care intensity on quality of life and symptom burden in patients undergoing palliative chemotherapy: A prospective cohort study
Source: Medicine (Baltimore). 2026 Jul 24;105(30):e49780. doi: 10.1097/MD.0000000000049780 (PMC13406126; doi:10.1097/MD.0000000000049780)
Supplement: Supplementary file 10 [file medi-105-e49780-s010.docx]

**Supplementary Table S10. Subgroup Analyses for Global QOL and ESAS (Mixed-Effects Models)**

| **Subgroup** | **QOL β (95% CI)** | **Pinteraction** | **ESAS β (95% CI)** | **Pinteraction** |
| --- | --- | --- | --- | --- |
| Age <65 (n=102) | 4.82 (2.01 to 7.63) | 0.412 | -2.45 (-3.92 to -0.98) | 0.365 |
| Age ≥65 (n=78) | 5.91 (2.34 to 9.47) |  | -1.98 (-3.55 to -0.41) |  |
| Male (n=94) | 5.12 (1.98 to 8.27) | 0.628 | -2.16 (-3.71 to -0.61) | 0.544 |
| Female (n=86) | 5.74 (2.44 to 9.04) |  | -2.36 (-4.01 to -0.71) |  |
| ECOG 0–1 (n=126) | 6.02 (3.11 to 8.93) | 0.287 | -2.88 (-4.31 to -1.44) | 0.091 |
| ECOG ≥2 (n=54) | 4.21 (0.91 to 7.51) |  | -1.14 (-3.06 to 0.78) |  |
| GI cancers (n=72) | 5.34 (2.11 to 8.56) | 0.852 | -2.41 (-4.02 to -0.81) | 0.903 |
| Lung cancer (n=48) | 5.89 (1.47 to 10.31) |  | -2.22 (-4.39 to -0.06) |  |
| Breast cancer (n=36) | 4.76 (0.92 to 8.60) |  | -1.96 (-3.88 to -0.04) |  |
| Hepatobiliary (n=24) | 6.10 (1.55 to 10.65) |  | -2.55 (-4.98 to -0.12) |  |
| Other tumors (n=20) | 5.01 (0.73 to 9.29) |  | -2.01 (-4.42 to 0.41) |  |

*No significant interaction effects were detected; associations remained directionally consistent.*
