# Supplementary material for: Impact of nurse-led supportive care intensity on quality of life and symptom burden in patients undergoing palliative chemotherapy: A prospective cohort study
Source: Medicine (Baltimore). 2026 Jul 24;105(30):e49780. doi: 10.1097/MD.0000000000049780 (PMC13406126; doi:10.1097/MD.0000000000049780)
Supplement: Supplementary file 11 [file medi-105-e49780-s011.docx]

**Supplementary Table S11. Sensitivity Analyses: Complete Case vs Multiple Imputation**

| **Outcome** | **Complete Case (n=134)** | **Multiple Imputation (n=180)** | **% Difference** |
| --- | --- | --- | --- |
| QOL β (95% CI) | 5.44 (2.91 to 7.96) | 5.62 (3.14 to 8.09) | +3.3% |
| QOL × Time p-value | 0.008 | 0.006 |  |
| ESAS β (95% CI) | -2.31 (-3.61 to -1.00) | -2.21 (-3.41 to -1.01) | -4.3% |
| ESAS × Time p-value | 0.014 | 0.011 |  |
| Logistic: QOL ≥10-point improvement, aOR (95% CI) | 1.42 (1.09 to 1.88) | 1.46 (1.12 to 1.90) | +2.8% |
| p-value | 0.009 | 0.006 |  |
| Logistic: Persistent High ESAS, aOR (95% CI) | 0.70 (0.53 to 0.94) | 0.71 (0.54 to 0.93) | +1.4% |
| p-value | 0.016 | 0.014 |  |

*Results were highly consistent across complete-case and MI methods.*
