# Supplementary material for: Impact of nurse-led supportive care intensity on quality of life and symptom burden in patients undergoing palliative chemotherapy: A prospective cohort study
Source: Medicine (Baltimore). 2026 Jul 24;105(30):e49780. doi: 10.1097/MD.0000000000049780 (PMC13406126; doi:10.1097/MD.0000000000049780)
Supplement: Supplementary file 12 [file medi-105-e49780-s012.docx]

**Supplementary Table S12. Sensitivity Analysis Excluding Early Deaths and Heavy Dropouts**

| **Outcome** | **Estimate (95% CI)** | **p-value** |
| --- | --- | --- |
| QOL (LMM β) | 5.71 (3.18 to 8.24) | <0.001 |
| ESAS (LMM β) | -2.34 (-3.78 to -0.90) | 0.001 |
| Logistic: QOL ≥10-point improvement, aOR (95% CI) | 1.52 (1.14 to 2.03) | 0.004 |
| Logistic: Persistent High ESAS, aOR (95% CI) | 0.69 (0.51 to 0.92) | 0.011 |

*Excluding early deaths and heavy dropouts did not materially change the findings.*
