# Supplementary material for: Impact of nurse-led supportive care intensity on quality of life and symptom burden in patients undergoing palliative chemotherapy: A prospective cohort study
Source: Medicine (Baltimore). 2026 Jul 24;105(30):e49780. doi: 10.1097/MD.0000000000049780 (PMC13406126; doi:10.1097/MD.0000000000049780)
Supplement: Supplementary file 13 [file medi-105-e49780-s013.docx]

**Supplementary Table S13. Follow-up and Event Overview**

| **Item** | **Value** |
| --- | --- |
| Median follow-up time, months | 14.8 (IQR 9.6–22.3) |
| Total deaths during follow-up | 58 (32.2%) |
| All-cause mortality rate (per 100 PY) | 28.7 |
| Loss to follow-up | 21 (11.7%) |
| Censoring rate | 37.2% |
| Participants with complete survival data | 159 (88.3%) |

Note: PY = person-years. Survival time measured from enrollment to death or last known contact.
