# Supplementary material for: Impact of nurse-led supportive care intensity on quality of life and symptom burden in patients undergoing palliative chemotherapy: A prospective cohort study
Source: Medicine (Baltimore). 2026 Jul 24;105(30):e49780. doi: 10.1097/MD.0000000000049780 (PMC13406126; doi:10.1097/MD.0000000000049780)
Supplement: Supplementary file 14 [file medi-105-e49780-s014.docx]

**Supplementary Table S14. Univariate Cox Regression for Overall Mortality**

| **Variable** | **HR (95% CI)** | **p-value** |
| --- | --- | --- |
| Supportive Care Intensity (per 1-point) | 0.91 (0.86–0.97) | 0.002 |
| High SCI (Q4 vs Q1–Q3) | 0.72 (0.51–1.02) | 0.064 |
| Age (per 10-year increase) | 1.18 (1.02–1.36) | 0.024 |
| Male sex | 1.21 (0.77–1.89) | 0.400 |
| ECOG ≥2 | 1.87 (1.25–2.80) | 0.003 |
| Baseline ESAS (per 5 points) | 1.09 (1.05–1.14) | <0.001 |
| Baseline global QOL (per 10 decrease) | 1.14 (1.03–1.26) | 0.011 |
| Cancer type: GI vs others | 1.32 (0.87–2.00) | 0.192 |
| HADS depression ≥8 | 1.41 (1.01–1.98) | 0.046 |

Note: HR < 1 indicates lower mortality risk.
