# Supplementary material for: Impact of nurse-led supportive care intensity on quality of life and symptom burden in patients undergoing palliative chemotherapy: A prospective cohort study
Source: Medicine (Baltimore). 2026 Jul 24;105(30):e49780. doi: 10.1097/MD.0000000000049780 (PMC13406126; doi:10.1097/MD.0000000000049780)
Supplement: Supplementary file 15 [file medi-105-e49780-s015.docx]

**Supplementary Table S15. Threshold Cox Model Based on Number of Nurse-Led Contacts**

| **Variable** | **Adjusted HR (95% CI)** | **p-value** |
| --- | --- | --- |
| ≥8 contacts vs <8 contacts | 0.63 (0.44–0.90) | 0.010 |
| Age (per 10-year increase) | 1.20 (1.04–1.39) | 0.014 |
| Male sex | 1.18 (0.75–1.86) | 0.470 |
| ECOG ≥2 | 1.72 (1.13–2.62) | 0.012 |
| Cancer type: GI vs others | 1.29 (0.83–1.99) | 0.253 |
| Baseline global QOL (per 10 decrease) | 1.11 (1.00–1.24) | 0.051 |
| Baseline ESAS (per 5 points) | 1.07 (1.03–1.12) | <0.001 |

Note: ≥8 contacts was defined a priori as “high-frequency supportive care.”
