# Supplementary material for: Impact of nurse-led supportive care intensity on quality of life and symptom burden in patients undergoing palliative chemotherapy: A prospective cohort study
Source: Medicine (Baltimore). 2026 Jul 24;105(30):e49780. doi: 10.1097/MD.0000000000049780 (PMC13406126; doi:10.1097/MD.0000000000049780)
Supplement: Supplementary file 16 [file medi-105-e49780-s016.docx]

**Supplementary Table S16. Exploratory Pathway Associations Involving Early QOL Change and Mortality**

| Pathway Component | Estimate (95% CI) | p-value |
| --- | --- | --- |
| Higher SCI → early QOL improvement (ΔQOL at 18 weeks) | β = 0.42 (0.23–0.61) | <0.001 |
| Early QOL improvement → subsequent mortality (per 10-point increase) | HR = 0.78 (0.66–0.92) | 0.004 |
| SCI → mortality, additionally adjusted for early QOL improvement | HR = 0.74 (0.51–1.05) | 0.089 |
| SCI → mortality, without additional adjustment for early QOL improvement | HR = 0.68 (0.48–0.96) | 0.030 |
| Attenuation in the SCI–mortality association after adjustment for early QOL improvement | Descriptive change in HR estimates | – |

*Interpretation: Higher supportive care intensity was associated with greater early improvement in global QOL, and early QOL improvement was in turn associated with lower subsequent mortality risk. The attenuation of the SCI–mortality association after additional adjustment for early QOL improvement is descriptive and should not be interpreted as evidence of indirect effects or causal mediation.*
